# Preliminary Characterization of Dog Derived Pathogenic Strains of *Leptospira interrogans* Serovar Australis in Nanchang of Jiangxi Province, China

**DOI:** 10.3389/fvets.2020.607115

**Published:** 2021-01-14

**Authors:** Ning Song, Wenlong Zhang, Yue Ding, Dianjun Wu, Zonghao Dai, Li Xu, Yongguo Cao

**Affiliations:** ^1^Department of Clinical Veterinary Medicine, College of Veterinary Medicine, Jilin University, Changchun, China; ^2^Nanchang Police Dog Base of the Ministry of Public Security, Nanchang, China

**Keywords:** *Leptospira interrogans* Serovar Australis, PCR, MAT, MLST (multilocus sequence typing), dog, hamster

## Abstract

Leptospirosis is a global zoonotic disease caused by pathogenic *Leptospira*, and those infected animals will show a variety of clinical symptoms and even death. The discovery of endemic strains is crucial to produce effective vaccines. In this study, we report that a strain of *Leptospira*, isolated from a dog, is pathogenic. Using MLST analysis, the serovar of isolated *Leptospira* was identified and found it belongs to *Leptospira interrogation* Serovar Australis. Then, the virulence of this strain was researched by using hamsters. After infection, all the hamsters died within 4–5 days. Typical pathological changes were found in the liver, kidney, and lung of hamsters. These results all indicated that the isolated *Leptospira* was pathogenic. Thus, this study facilitates to identifying local *Leptospira* strains and develop a more targeted canine *Leptospira* vaccine.

## Introduction

Leptospirosis, caused by a pathogenic *Leptospira*, is a zoonotic disease. Human may get leptospirosis directly or indirectly from animals (1). Leptospirosis occurs worldwide, especially in tropical and subtropical regions where rains a lot. On a global scale, it is conservatively estimated that ~500,000 serious cases occur each year, with a case fatality rate ranging from 3 to 70%, depending on severity of clinical symptoms (2, 3). Preliminary results indicate that the currently incidence of Leptospirosis may give rise to a serious underestimation of actual cases. This underestimation is partly due to the no characteristic manifestations in Leptospirosis, which are often confused with other diseases that is prevalent under similar environmental and climatic conditions, such as dengue fever, rickettsia disease, enteritis, and malaria (4).

The first Leptospirosis case report was in 1934 in mainland China. So far, more than 2.5 million people have been infected in China, more than 20,000 people died from Leptospirosis, and at least 80% of cases have occurred from July to December. Because of the climatic factors, September is the peak season during this period:floods are common accompanied by severe weather, such as typhoons (5). Animal Leptospirosis is mainly concentrated in the south of China. In recent years, the domestic pet industry has developed rapidly, which has led to a significant increase in the risk of canine infection with Leptospirosis (6). In the four representative cities in Jiangxi province (Nanchang, Yichun, Shangrao, and Ganzhou), the blood positive rate of stray dogs and pet dogs reached 4.79 and 1.97%, respectively. Because dogs have a closer relationship with humans than economic animals such as pigs and cattle, *Leptospira* is likely to infect humans through them and pose a serious public health hazard. In addition to human hosts, pathogenic *Leptospira* also infects a variety of animals, including domestic mammals (livestock) and wild animals, especially rodents, which are considered to be the main source of human *Leptospira* infection (7). Humans can be infected through direct contact with infected animals, or indirect contact with water and soil contaminated by the urine of infected animals (8). Since it was first confirmed by serology and etiology in Gan County, Jiangxi Province in 1958, it occurred or spread to varying degrees every year.

In recent years, the typing method based on multi-site sequence analysis has gradually been applied to bacteriological genetyping and molecular epidemiological research. The dog, we studied in this study, has clinical symptoms of vomiting, loss of appetite, and jaundice, which is like the symptoms of Leptospirosis. Therefore, the dog was subjected to blood routine, biochemical examination (Table 1), and serological examination (MAT). However, the sick dog failed to recover and died. In this study, experimental techniques such as MAT, PCR, and MLST were mainly used to identify *Leptospira* isolated from the sick dog and determine its molecular type and serovar. We also preliminary studied the virulence of this strain, which provided a basis for the prevention and control of *Leptospira*. This indicates that the *Leptospira* that is prevalent in dogs in Nanchang maybe this strain. It also helps to develop a canine vaccine against the endemic *Leptospira*, effective prevention of Leptospirosis from dogs to humans.

**Table 1:** Blood routine and biochemical test results.

**Test item**	**Test result**	**Reference range**
RBC	6.75 × 10^12^/L	5.50–8.50
HCT	44.80%	37.0–55.0
HGB	16.6 g/Dl	12.0–18.0
MCV	66.4 fL	60.0–77.0
MCH	24.5 pg	18.5–30.0
MCHC	36.9 g/Dl	30.0–37.5
RDW	16.60%	14.7–17.9
%RETIC	0.80%	
RETIC	53.3 K/μL	10.0–110.0
WBC	27.18 × 10^9^/L	5.50–16.90
%NEU	74.10%	
%LYM	7.80%	
%MONO	16.30%	
%EOS	1.50%	
%BASO	0.30%	
NEU	20.15 × 10^9^/L	2.00–12.00
LYM	2.12 × 10^9^/L	0.50–4.90
MONO	4.42 × 10^9^/L	0.30–2.00
EOS	0.41 × 10^9^/L	0.10–1.49
BASO	0.08 × 10^9^/L	0.00–0.10
PLT	301 K/ml	175–500
MPV	4.2 fL	
PDW	21.90%	
PCT	0.12%	
UREA	38.2 mmol/L	2.5–9.6
CREA	192 μmol/L	44–159
PHOS	4.25 mmol/L	0.81–2.19
ALT	598 U/L	10–100
AST	82 U/L	0–50

## Materials and Methods

### Case Description

A 3 year-old female mixed breed dog was sent to the Nanchang Police Dog Base Animal Hospital for treatment. According to the owner of the sick dog, the dog lacked energy with decreased appetite, vomiting, and yellow urine. The course of the disease is about 5 days. Temperature, heart rate, and respiratory rate were recorded at 39.8°C, 130, and 40 bpm, respectively. Icteric mucous membranes were found in the dog by the clinical examination, and the color of the serum was dark yellow. The complete blood count and serum biochemistry showed an increase in WBC, NEU, and MONO, CREA, UREA, PHOS, ALT, and AST, indicating serious problems with liver and kidney function (Table 1). Fluid therapy was initiated immediately through an intravenous catheter. However, the sick dog failed to recover and died. With the consent of the owner, we performed an autopsy and collected its serum, liver, and kidneys.

### Ethics Statement

During the experiment, in a 12 h light and a 12 h dark cycle, hamsters drank freely with standard rodent food. Hamsters experiments were performed according to regulations of the Administration of Affairs Concerning Experimental Animals in China. Due to the serious condition of the dog, it eventually fell ill and died. With the consent of the owner, we performed an autopsy and collected its serum, liver, and kidneys. All the protocol include hamsters and dog (Autopsy of the cadaver and sample collection), was approved by the Institutional Animal Care and Use Committee of Jilin University (20170318).

### Bacterial Strain Isolation, Cultivation, and Animals

All the strains of serotype pathogenic *Leptospira interrogans* and *L. biflexa* serovar Patoc were cultivated in Ellinghausen–McCullough–Johnson–Harris (EMJH) medium at 29°C for 5–7 days to observe bacterial growth (9). Before use, bacterial concentration was determined using a Petroff–Hausser counting chamber. The count was performed at 400x magnification under a darkfield, and DNA was extracted when the number of *Leptospira* reached 2 × 10^8^ cells/ml.

The liver and kidney of dog were disinfected with 75% alcohol. Then the kidney cortex and liver parenchymal tissue pieces (about the size of a rice grain) were cut out in a clean bench and cultured in EMJH medium for 5–7 days to observe bacterial growth. When *Leptospira* was observed in darkfield, EMJH solid medium was prepared, and the bacterial diluent was smeared on the solid medium for cultivation. Later, single colonies that had grown were picked and inoculated in the EMJH medium for culture. Syrian golden hamsters (Mesocricetus auratus) were provided by the Animal Center of Jilin University.

### Preparation of DNA

When the number of *Leptospira* reached 2 × 10^8^ cells/ml, 1.2 ml of 15 pathogenic *Leptospira*, the newly isolated *Leptospira, L. biflexa serovar* Patoc and were taken and centrifuged at 12,000 rpm for 10 min. Then the supernatant was discarded to obtain a precipitate. The DNA was then extracted using the TIAN-amp DNA Minikit.

### Primer Design

The primer was designed by the laboratory with an annealing temperature of 60°C. The GeneBank database was used to query and download the published sequence of the common pathogenic *Leptospira* LipL32 gene. All the sequences obtained were analyzed and compared by DNAMAN software to select a highly conserved sequence among them. Primers were designed using the biological software Primer Premier 6.0 according to the principle of primer design. To ensure the high specificity of the primers involved, all involved primer sequences were validated in the NCBI database. The sequence was sent to Comate Bioscience Company Limited for synthesis, and after centrifugation according to the instructions, the corresponding volume of TE was added for dissolution. The size of the target fragment was 468 bp. The forward primer was 5′-CRGGCGACGGAGAYTTAG-3′; The downstream primer was 5′-AGAYTCTTCRGCDGCKATRG-3′.

### PCR Amplification

The PCR program for the primers was as follows: predenaturation at 94 °C for 3 min; denaturation at 94°C for 30 s, annealing at 60°C for 30 s, extension at 72°C for 30 s for 35 cycles, and an extension at 72°C for 5 min. The reaction volume was 25 μl and consisted of 1 μl of each primer, 1 μl of DNA template,12.5 μl of Taq, and 5.5 μl of sterile water. After PCR run, the newly isolated *Leptospira* sample's PCR production was sequenced. Then the amplification sequences were comparative with outer membrane protein (lipL32) gene of *Leptospira*.

### MLST Analysis

MLST website was established to provide public access to these data and to provide the resource to other investigators who can use this to assign the ST of further strains. This was accessed at http://leptospira.mlst.net. DNA was extracted from the cultures of the *Leptospira* by using the TIAN-amp DNA Minikit. Based on other literature, seven locis (pntA, sucA, caiB, tpiA, mreA, pfkB, and glmU) were used for MLST (10). Primer sequences were showed in Table 3. Amplifications were performed in a 25 μl reaction system containing 1 μL (1 ng/μL) of genomic DNA, 1 μl of each primer (0.2 μmol). PCR was performed with an initial denaturing step at 94°C for 5 min, followed by 30 cycles of 94°C for 10 s, 52°C (mreA, pfkB, pntA, sucA, and tpiA), or 50°C (caiB and glmU) for 15 s, 72°C for 50 s, then 72°C for 7 min. PCR product size ranged from 555 to 638 bp. MLST was performed for the isolates by using these 7 loci. Following the standard MLST protocol, each allele was assigned a different allele number and the allelic profile (a string of seven integers) was used to define the sequence type (ST).

### Infection in the Hamster Model and Histopathological Examination

To determine whether the isolated strain was pathogenic, four 21 day-old hamsters were inoculated intraperitoneally with ~10^8^ cells/ml of this strain (11), four were inoculated 10^7^ cells/ml, four were inoculated 10^6^ cells/ml, the other four were injected with an equal volume of saline as control group. All of the hamsters were monitored daily for the appearance of clinical signs. Necropsy was performed when the hamster dead. Kidneys, lung and liver were aseptically removed, macerated and suspended in a liquid medium for re-isolation. Organs for histopathological examination were fixed in 10% formalin for 48–72 h, dehydrated in gradient alcohol series, embedded in paraffin, and stained with hematoxylin and eosin (H&E) (12).

### Serology

Currently, MAT is one of the most important diagnostic tests and it is commonly used as a serological gold standard (13). Firstly, we performed MAT qualitative experiments on the serum of the sick dog, and evaluated the titer after confirming its serology. Secondly, we injected this strain into the abdominal cavity of four 3 week-old golden hamsters at 2 × 10^8^ cells/ml. Before the experimental group of hamsters died, blood was collected from the ocular nerve plexus, and the serum was collected by centrifugation for MAT.

## Results

### Clinical Symptoms of a Sick Dog and Bacterial Isolation

**Figure 1:**
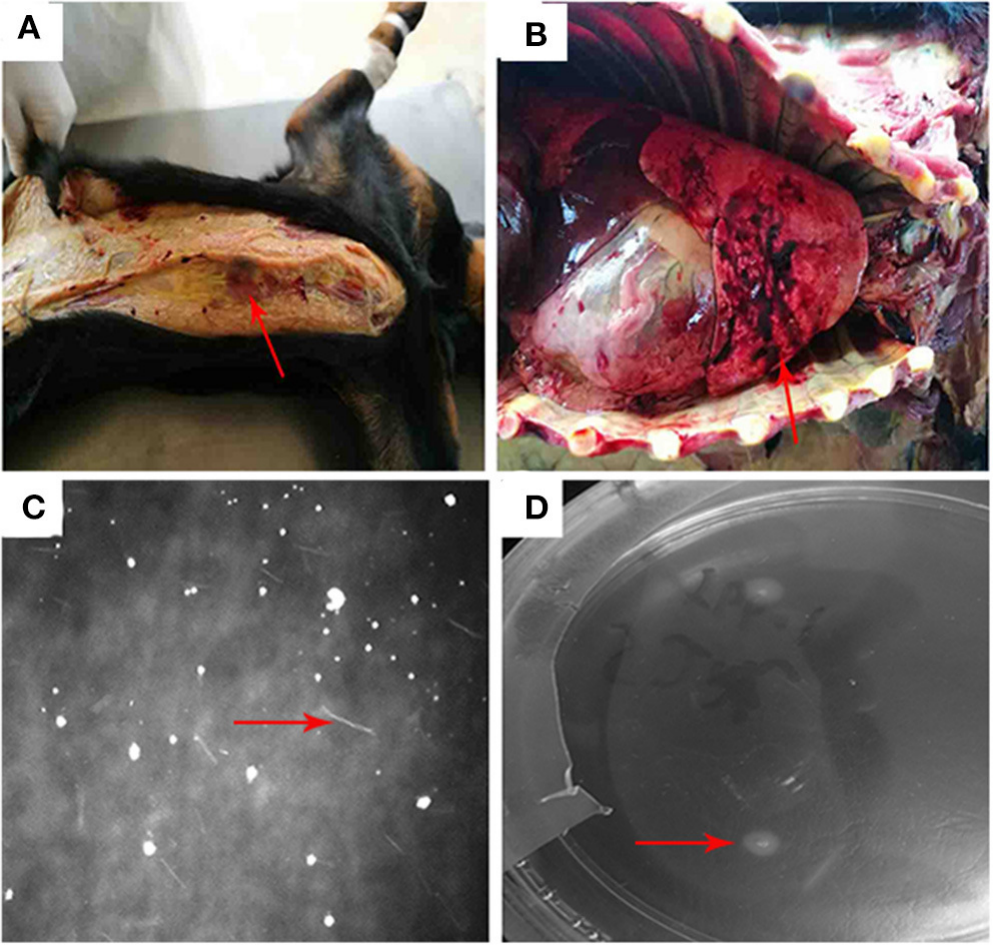
**(A)** The dog was found to have jaundice. **(B)** hemorrhage from the lungs was relatively serious: **(C)** *Leptospira* in liver and kidney cultures were observed under a dark field microscope. **(D)** a single colony of *Leptospira* was observed on the EMJH solid medium.

At autopsy, the dog was found to have jaundice (Figure 1A). Hemorrhage in lung was severe (Figure 1B). No obvious abnormalities were found in the liver and kidneys. At the 10th day of culture, the *Leptospira* in liver and kidney were observed under a dark-field microscope (Figure 1C). At the 15th day of culture, a single colony of *Leptospira* was observed on the EMJH solid medium (Figure 1D).

### The Reliability of the Primer

**Figure 2:**
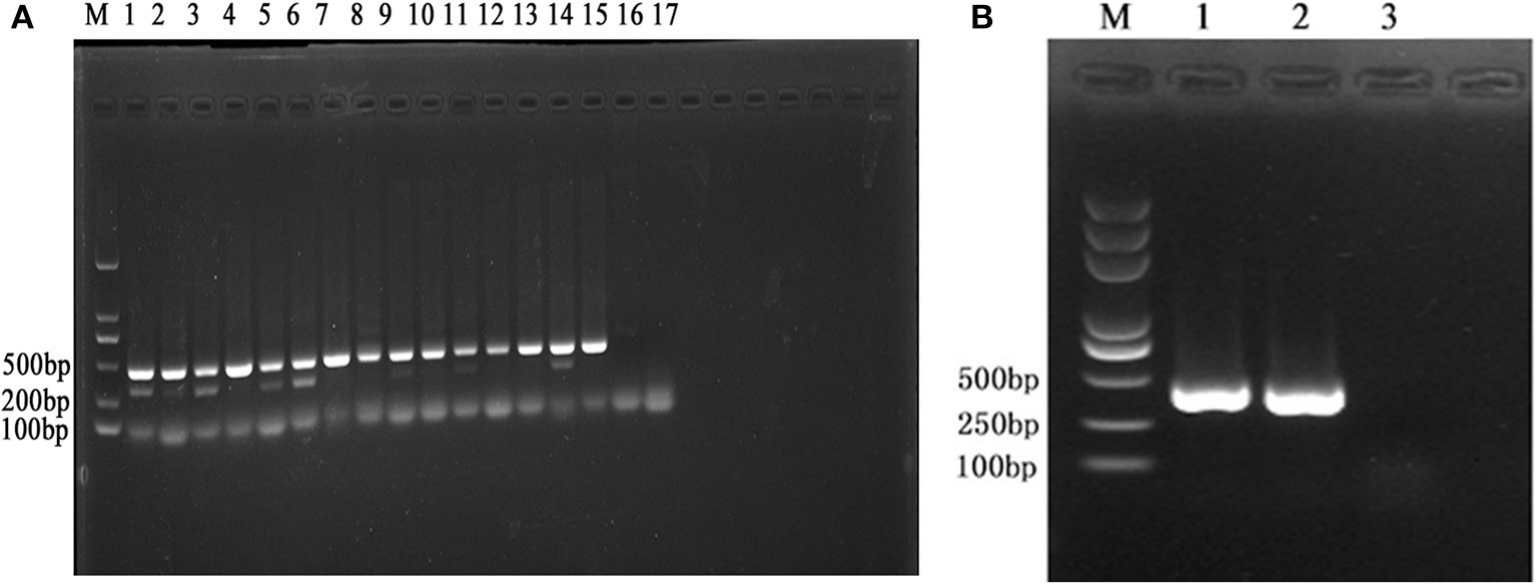
**(A)** M is expressed as DNA maker, 1–17 is expressed as *Leptospira* standard strain Lai 56601, Javanica 56602, Canicola 56603, Ballum 56604, Pyrogenes 56605, Autumnalis 56606, Australis 56607, Pomona 56608, Linhai 56609, Hebdomadis 56610. Paidjan 56612, Tarasovi 56613, Cingshui 56615, Wulffi 56635, Mini 56655, *L. biflexa serovar* Patoc, negative control. **(B)** M is expressed as DNA maker, 1–3 is expressed as: *Leptospira* standard strain Lai 56601, sample, negative control.

To analyze the reliability of the primer, the DNA of 15 pathogenic *Leptospira* serovar and Patoc was extracted and their concentrations were determined (Table 2). Then, PCR was performed to observe the band's condition (Figure 2A). All the 15 pathogenic *Leptospira* Serovar were detected. All experiments were conducted more than three times.

**Table 2:** *Leptospira* strains used in this study.

**Serotype and strain**	**Species**	**Status**	**Concentration ng/μl**	**Result**
Lai 56601	*L. interrogans*	Pathogenic	3.95	+
Javanica 56602	*L. interrogans*	Pathogenic	6.08	+
Canicola 56603	*L. interrogans*	Pathogenic	3.25	+
Ballum 56604	*L. interrogans*	Pathogenic	7.55	+
Pyrogenes 56605	*L. interrogans*	Pathogenic	1.85	+
Autumnalis 56606	*L. interrogans*	Pathogenic	3.6	+
Australis 56607	*L. interrogans*	Pathogenic	1.05	+
Pomona 56608	*L. interrogans*	Pathogenic	44	+
Linhai 56609	*L. interrogans*	Pathogenic	0.7	+
Hebdomadis 56610	*L. interrogans*	Pathogenic	46.2	+
Paidjan 56612	*L. interrogans*	Pathogenic	0.75	+
Tarasovi 56613	*L. interrogans*	Pathogenic	91.8	+
Cingshui 56615	*L. interrogans*	Pathogenic	5.25	+
Wulffi 56635	*L. interrogans*	Pathogenic	1.75	+
Mini 56655	*L. interrogans*	Pathogenic	26.05	+
Patoc	*L. biflexa*	Non-pathogenic	12.6	–
Negative control (water)				–

**Table 3:** Information of Loci Proposed for MLST of *Leptospira*l Isolates.

**Gene**	**Size of PCR Product (bp)**	**Primer 5^**′**^-3^**′**^**	**Annealing** **temperature** **(**^**°**^**C)**
CaiB	650	CAACTTGCGGAYATAGGAGGAG	46
		ATTATGTTCCCCGTGAYTCG	
PntA	638	TGCCGATCCTACAACATTA	52
		AAGAAGCAAGATCCACAACTAC	
SucA	560	AGAAGAGGCCGGTTATCATCAG	52
		CTTCCGGGTCGTCTCCATTTA	
PfkB	560	CCGAAGATAAGGGGCATACC	52
		CAAGCTAAAACCGTGAGTGATT	
TpiA	534	AAGCCGTTTTCCTAGCACATTC	52
		AGGCGCCTACAAAAAGACCAGA	
MreA	602	AAAGCGGCCAACCTAACACC	52
		CGATCCCAGACGCAAGTAAG	
GluM	557	GGAAGGGCACCCGTATGAA	50
		TCCCTGAGCGTTTTGATTT	

### The Newly Isolated *Leptospira* Sample Detection

The results showed that a band appears at 468 bp,it was *Leptospira interrogans* (Figure 2B). The sequence comparative analysis result shows it was *Leptospira interrogans*. The amplification sequences were showed in Table 4. All experiments were conducted more than three times.

**Table 4:** The newly isolated *Leptospira* sample PCR production amplification sequences (436 bp).

ATAGCTTGTTTTTGCAATTCTTCAGGATTTGAGTGGATCAGCGGGCT CACACCTGGAATACCTGGTGGAAAAAGCAGACCAACAGATGCAAC GAAAGATCCTTTCACTTCACCTGGTTTGTAGGTAGTGAAAGAAATTC TGTAAAGACCTCTTACTAAAAGTTTTTTAGTGTCGATGTTTTTCAGAT CGTCAAAAGATTTTGGAGGATTAGGGATCTTGATTCTAGTAAGAGAG TTGTACTTGTTGTGTCTCTCTTCTTTATAAGTATCGTCACCATCATCA TCATCGTCTAATTTTTGAACTGGTTTTGCTTTCGCAGCTTTGGCGATT TGGTCAGGCATAATCGCCGACATTCTTTCTACACGGATCCAAGTAT CAAACCAATGTGGCATTGATTTTTCTTCTGGGGTAGCCGCTTTGAAA GCGTCGCTTACTA

### MLST Result

All the seven locis were successfully amplified from the isolate *Leptospira*. MLST was performed for the isolate by using the 7 loci. Following the standard MLST protocol which was accessed at http://leptospira.mlst.net, an allele number was assigned to all the allele of different *Leptospiral* strains and the allelic profile was defined as sequence type (ST) 93(a string of seven allele number for the single colony of *Leptospira* was 1-2-3-5-6-6-7). According to the ST profile, the *Leptospiral* strains were defined as *Leptospira interrogans* Serovar Australis.

### Infection and Histopathological Examination Result

**Figure 3:**
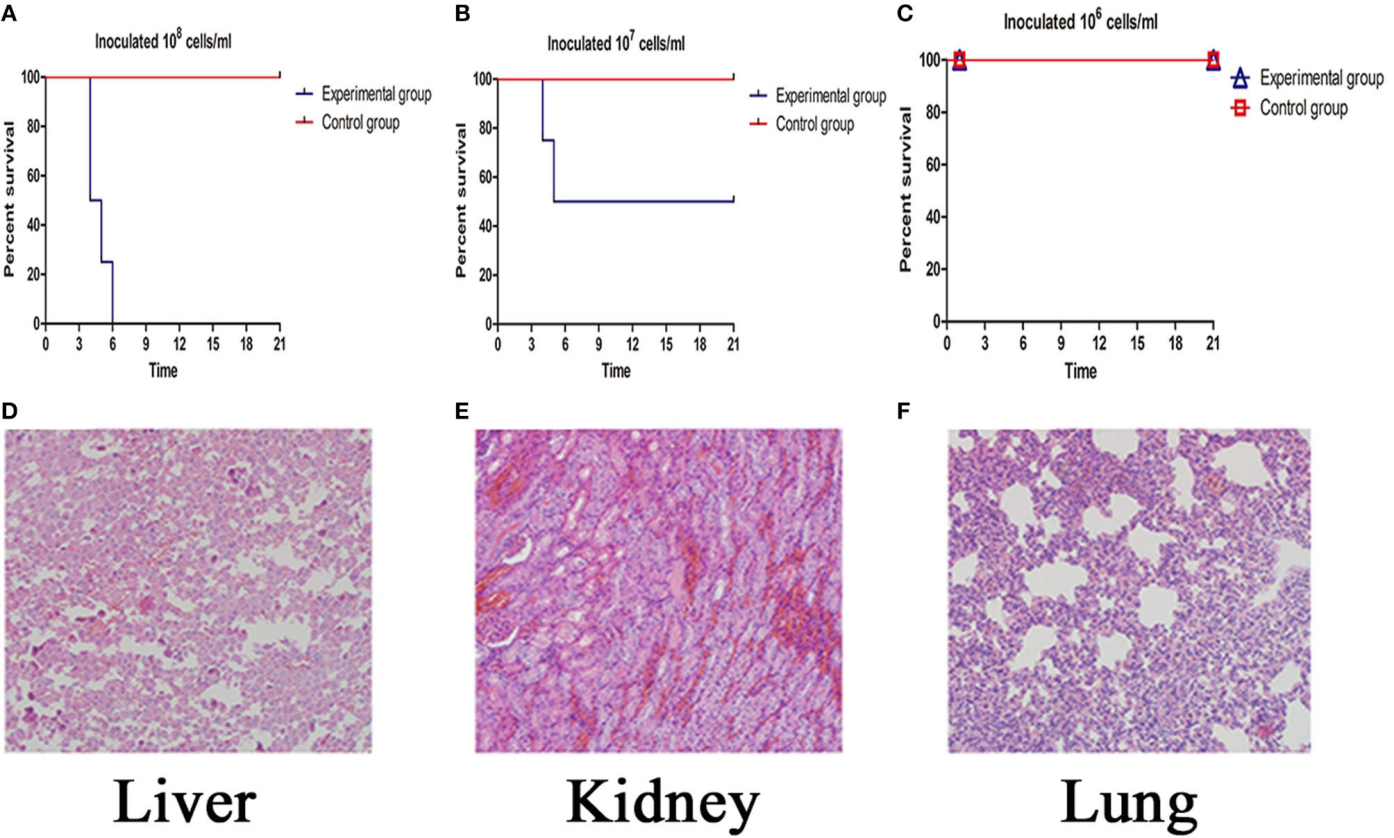
**(A–C)** The survival rate of the inoculated 10^8^-10^6^ cells/ml *Leptospira* experimental group. **(D–F)** Histopathology of the liver (200×), kidney (200×), lung (200×).

After infection, the isolated strain caused clinical symptoms, including dehydration, fluffy hair, poor mental state, and curled into the corner alone. All the hamsters in the group of inoculated 10^8^ cells/ml of this strain dead (Figure 3A) at the fourth day after infecton. Two hamsters in the group of inoculated 10^7^ cells/ml died quickly on the fourth and fifth days (Figure 3B). However, all hamsters in the group of inoculated 10^6^ cells/ml were alive (Figure 3C). Pathological changes in the livers of infected animals were distinct with increased permeability of hepatic tight junctions and areas of necrosis and inflammatory infiltration. Meanwhile, the renal tissues of the infected group showed a dramatic lesion with hemorrhage. We also found that generalized interstitial pneumonia was noted in the lungs of the infected group, with hemorrhagic areas, alveolar congestion, and infiltrate of mononuclear cells (Figures 3D–F).

### Serology

After MAT, serum from the sick dog had the most obvious anti-serum agglutination with *Leptospira interrogans* Serovar Australis, whose Strain number is 56607 (Table 5). Serum from the infected hamster showed the same result as the dog, further proving that the isolated strain was *Leptospira interrogans* Serovar Australis (Table 6).

**Table 5:** Anti-*Leptospira* serum of dog by MAT.

**Serogroup**	**Strain**	
Icterohaemorrhagiae	56601	–
Javanica	56602	–
Canicola	56603	+(1:100)
Ballum	56604	+(1:200)
Pyrogenes	56605	–
Autumnalis	56606	–
Australis	56607	++++(1:6400)
Pomona	56608	–
Grippotyphosa	56609	–
Hebdomadis	56610	–
Bataviae	56612	–
Manhao	56615	–
Sejroe	56635	–
Mini	56655	–

**Table 6:** Anti-*Leptospira* serum of hamster by MAT.

**Serogroup**	**Strain**	**Control** >**group**	**Experimental** >**group**
Icterohaemorrhagiae	56601	–	–
Javanica	56602	–	–
Canicola	56603	–	–
Ballum	56604	–	+(1:50)
Pyrogenes	56605	–	–
Autumnalis	56606	–	–
Australis	56607	–	++(1:1600)
Pomona	56608	–	–
Grippotyphosa	56609	–	–
Hebdomadis	56610	–	–
Bataviae	56612	–	–
Manhao	56615	–	–
Sejroe	56635	–	–
Mini	56655	–	–

## Discussion

*Leptospira* has a long and thin spiral shape and a more active state of motion can be observed under the microscope. Its diameter is approximately about 0.2 μm, and the length ranges from 5 to 25 μm (14). The outer membrane contains lipopolysaccharide, like the genus *Pseudomonas* but not to *Borrelia* and *Treponema pallidum*. The main pathogenic *Leptospira* is mainly *Leptospira interrogans*. In this study, we showed that *Leptospira* isolated from the kidney of a dog in Nanchang, Jiangxi Province belonged to *Leptospira interrogans* Serovar Australis by PCR, MLST, and MAT analysis. The isolate strain was virulent by using the hamster model. Our results facilitated the active surveillance of Leptospirosis, local investigations, and source tracking.

Jiangxi province is an ancient source of Leptospirosis. Every year several cases of Leptospirosis are reported in Jiangxi provinces. From 2016 to 2018, 354, 201, and 157 cases of Leptospirosis were reported nationwide (excluding Hong Kong, Macao and Taiwan regions), with reported incidences of 0.0258/100,000, 0.0146/100,000, and 0.0113/100,000, respectively. From 2016 to 2018, Jiangxi Province reported a total of 19, 21 and 9 cases, with reported incidences of 0.0416/100,000, 0.0261/100,000, and 0.0195/100,000, respectively. The *Leptospira icterohaemorrhagiae* is the dominating epidemic serovar in Jiangxi Province, followed by the *Javanica*. *L. introrogans* is the main pathogenic gene species. ST1 is the main dominant ST type, followed by ST143. It is reported that *L. introrogans* gene species are more suitable for living in humid environments, while the *L. bergerpersenii* gene species can survive in both wet and dry conditions (15).

Multi-locus sequence typing (MLST) is an analysis method based on housekeeping gene sequence determination, which is used for strains genomic correlation and genetic diversity. MLST is a typing method established in recent years (16). It is based on the MLEE principle and a typing method of the nucleotide sequence of pathogenic bacteria. MLST is the product of a combination of high-throughput sequencing technology and mature population genetics. By analyzing the nucleotide sequences of core fragments of multiple housekeeping genes, 7–8 housekeeping genes were sequenced (17, 18). MLST is based on DNA sequencing data, which can make it specific, repeatable, and comparable, and it is also suitable for detecting strain evolution and genetic analysis of microbial populations (18). This method has high-resolution and good-reproducibility between different laboratories (19). The combined use of MLST and MAT will identify *Leptospira* more accurately (20).

Up to now, the epidemic situation of *Leptospira* in Nanchang has rarely been reported in China. In this study, we have characterized a pathogenic *Leptospira*, which belongs to the *Leptospira interrogans* serovar Australis. Further characterization of this isolate is underway and may help to understand the virulence and pathogenic mechanisms of *Leptospira* spp. We will also conduct a local epidemiological investigation to determine the prevalence of Leptospirosis and the strains. These efforts will have a positive effect on the prevention and control of Leptospirosis in the local area, and contribute to the preparation of a canine vaccine against the local epidemic of *Leptospira*.

## Data Availability Statement

The raw data supporting the conclusions of this article will be made available by the authors, without undue reservation.

## Ethics Statement

The animal study was reviewed and approved by Institutional Animal Care and Use Committee of Jilin University. Written informed consent was obtained from the owners for the participation of their animals in this study.

## Author Contributions

YC: conceptualization. NS and WZ: data curation. WZ: formal analysis and project administration. YC and LX: funding acquisition and writing—review and editing. NS and YD: investigation. NS and DW: methodology. DW and YD: software. YD: visualization. ZD: supervision. NS: writing—original draft.

## Conflict of Interest

The authors declare that the research was conducted in the absence of any commercial or financial relationships that could be construed as a potential conflict of interest.

## References

[1] Adler B de la Peña Moctezuma A. *Leptospira* and Leptospirosis. Vet Microbiol. (2010) 140:287–96. 10.1016/j.vetmic.2009.03.012 19345023

[2] Romero EC Yasuda PH Human Leptospirosis: guidance for diagnosis, surveillance and control. Rev Inst Med Trop São Paulo. (2003) 45:292 10.1590/S0036-46652003000500015 14743663

[3] Søndergaard MM Tursunovic A Thye-Rønn P Bang JC Hansen IM. Leptospirosis-associated severe pulmonary hemorrhagic syndrome with lower back pain as an initial symptom. Am J Case Rep. (2016) 17:883–6. 10.12659/AJCR.900477 27881835 PMC5127633

[4] Ahmed A Engelberts MFM Boer KR Ahmed N Hartskeerl RA. Development and validation of a real-time PCR for detection of pathogenic *Leptospira* species in clinical materials. PLoS ONE. (2009) 4:e7093. 10.1371/journal.pone.0007093 19763264 PMC2740861

[5] Weilin H Xu'Ai L Jie Y *Leptospira* and leptospirosis in China. Curr Opin Infect Dis. (2014) 27:432–6. 10.1097/QCO.0000000000000097 25061933

[6] Harkin KR Roshto YM Sullivan JT. Clinical application of a polymerase chain reaction assay for diagnosis of Leptospirosis in dogs. J Am Vet Med Assoc. (2003) 222:1224–9. 10.2460/javma.2003.222.1224 12725309

[7] Haake DA Levett PN. Leptospirosis in humans. Curr Top Microbiol Immunol. (2015) 387:65–97. 10.1007/978-3-662-45059-8_5 25388133 PMC4442676

[8] Ellis WA. Animal leptospirosis. Curr Top Microbiol Immunol. (2015) 387:99–137. 10.1007/978-3-662-45059-8_6 25388134

[9] Jin X Zhang W Ding Z Wang H Wu D Xie X. Efficacy of the rabbit polyclonal anti-*Leptospira* antibody against homotype or heterotype *Leptospira* infection in hamster. PLoS Negl Trop Dis. (2016) 10:e0005191. 10.1371/journal.pntd.0005191 28027297 PMC5189943

[10] Boonsilp S Thaipadungpanit J Amornchai P Wuthiekanun V Bailey MS Holden MTG. A Single Multilocus Sequence Typing (MLST) scheme for seven pathogenic *Leptospira* species. PLoS Negl. Trop Dis. (2013) 7:e1954. 10.1371/journal.pntd.0001954 23359622 PMC3554523

[11] Silva EF Santos CS Athanazio DA Seyffert N Seixas FK Cerqueira GM. Characterization of virulence of *Leptospira* isolates in a hamster model. Vaccine. (2008) 26:3892–6. 10.1016/j.vaccine.2008.04.085 18547690 PMC2519131

[12] Zhang W Zhang N Wang W Wang F Gong Y Jiang H. Efficacy of cefepime, ertapenem and norfloxacin against Leptospirosis and for the clearance of pathogens in a hamster model. Microbial Pathog. (2014) 77:78–83. 10.1016/j.micpath.2014.11.006 25450882

[13] Weiss S Menezes A Woods K Chanthongthip A Dittrich S Opoku-Boateng A. An extended Multilocus Sequence Typing (MLST) scheme for rapid direct typing of *Leptospira* from clinical samples. PLoS Negl Trop Dis. (2016) 10:e0004996. 10.1371/journal.pntd.0004996 27654037 PMC5031427

[14] Bharti AR Nally JE Ricaldi JN Matthias MA Diaz MM Lovett MA. Leptospirosis: a zoonotic disease of global importance. Lancet Infect Dis. (2003) 3:757–71. 10.1016/S1473-3099(03)00830-2 14652202

[15] Cosson JF Picardeau M Mielcarek M Tatard C Chaval Y Suputtamongkol Y. Epidemiology of *Leptospira* transmitted by rodents in Southeast Asia. PLoS Negl Trop Dis. (2014) 8:e2902. 10.1371/journal.pntd.0002902 24901706 PMC4046967

[16] Maiden MCJ Multilocus sequence typing of bacteria. Annu Rev Microbiol. (2006) 60:561–88. 10.1146/annurev.micro.59.030804.121325 16774461

[17] Feil EJ Enright MC. Analyses of clonality and the evolution of bacterial pathogens. Curr Opin Microbiol. (2004) 7:308–13. 10.1016/j.mib.2004.04.002 15196500

[18] Qi WH Lacher DW Bumbaugh AC Hyma KE Ouellette LM Large TM EcMLST: an online database for multi locus sequence typing of pathogenic Escherichia coli. In: 2004 IEEE Computational Systems Bioinformatics Conference, Proceedings. Stanford, CA, (2004) p. 520–21.

[19] Kaas RS Friis C Ussery DW Aarestrup FM. Estimating variation within the genes and inferring the phylogeny of 186 sequenced diverse *Escherichia coli* genomes. BMC Genomics. (2012) 13:577. 10.1186/1471-2164-13-577 23114024 PMC3575317

[20] Kanagavel M Margreat AAP Arunkumar M Prabhakaran SG Shanmughapriya S Natarajaseenivasan K. Multilocus sequence typing (MLST) of leptospiral strains isolated from two geographic locations of Tamil Nadu, India. Infect Genet Evol. (2016) 37:123–8. 10.1016/j.meegid.2015.11.008 26577860

