# Supplementary material for: Preliminary Characterization of Dog Derived Pathogenic Strains of Leptospira interrogans Serovar Australis in Nanchang of Jiangxi Province, China
Source: Front Vet Sci. 2021 Jan 14;7:607115. doi: 10.3389/fvets.2020.607115 (PMC7841341; doi:10.3389/fvets.2020.607115)
Supplement: Supplementary file 1 [file Data_Sheet_1.pdf]

Supplementary materials

The sensitivity and specificity of the primer

Specificity test

Listeria monocytogenes , *Staphylococcus aureus* , *Bacillus, Enterococcus faecalis, Lactobacillus*,*Escherichi coli* , *Legionella* ,*Bartonella, Phylum Apicomplexa* ,*Borrelia burgdorferi Eperythrozoon*,negative control have no specific bands, indicating specificity good(S1 Fig4A).

Sensitivity detection

The well-cultivated Lai 56601 strain was performed at 400x magnification under a darkfield. When the number of *Leptospira* reached 2 × 10^8 cells/ml, and diluted to 10^7 cells/ml, then extracte DNA. Ten microliters of the extracted DNA solution were pipetted into 90 μL of TE to prepare Leptospiral DNA solution of 10^6 and then diluted to 10^0 in a 10-fold gradient.

The results showed that the sensitivity of the primer for PCR was high, and the DNA content of one *Leptospira* was detected(S1 Fig4B).

Leptospiral burdens in the kidneys, livers, and lungs of hamsters

S1 Fig5 is Leptospiral burdens in the kidneys, livers, and lungs of hamsters in the infected group with 10^8 cells/ml.

S1:Fig4A: M is expressed as DNA maker, 1-10 is expressed as: *Leptospira* strain Lai 56601，*Listeria monocytogenes*, *Staphylococcus aureus, Bacillus, Enterococcus faecalis, Lactobacillus*, *Escherichia coli*, *Legionella, Bartonella,* negative control

FigB: The sensitivity of the primers for PCR. M is the DNA marker, and 1-6 indicates the concentration gradient of Lai 56601: 10^{6} -10^{0}.

Fig5 *Leptospiral* burdens in the kidneys, livers, and lungs of hamsters in the infected group with 10^8 cells/ml.

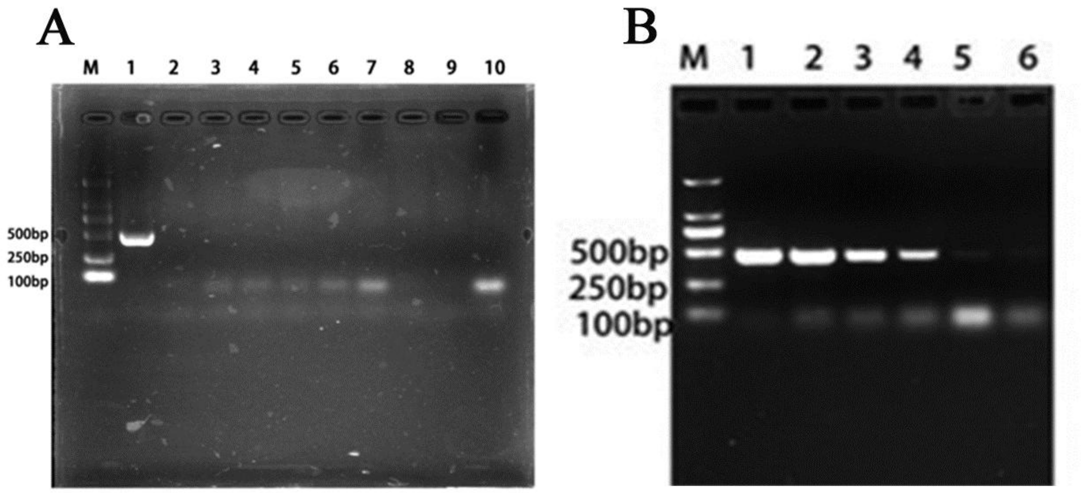

S1: Fig4**A:** M is expressed as DNA maker, 1-10 is expressed as: *Leptospira* standard strain Lai 56601 ， *Listeria monocytogenes*, *Staphylococcus aureus, Bacillus, Enterococcus faecalis, Lactobacillus*, *Escherichia coli*, *Legionella, Bartonella,* negative control **B:** The sensitivity of the primers for PCR. M is the DNA marker, and 1-6 indicates the concentration gradient of Lai 56601: 10^{6} -10^{0}.

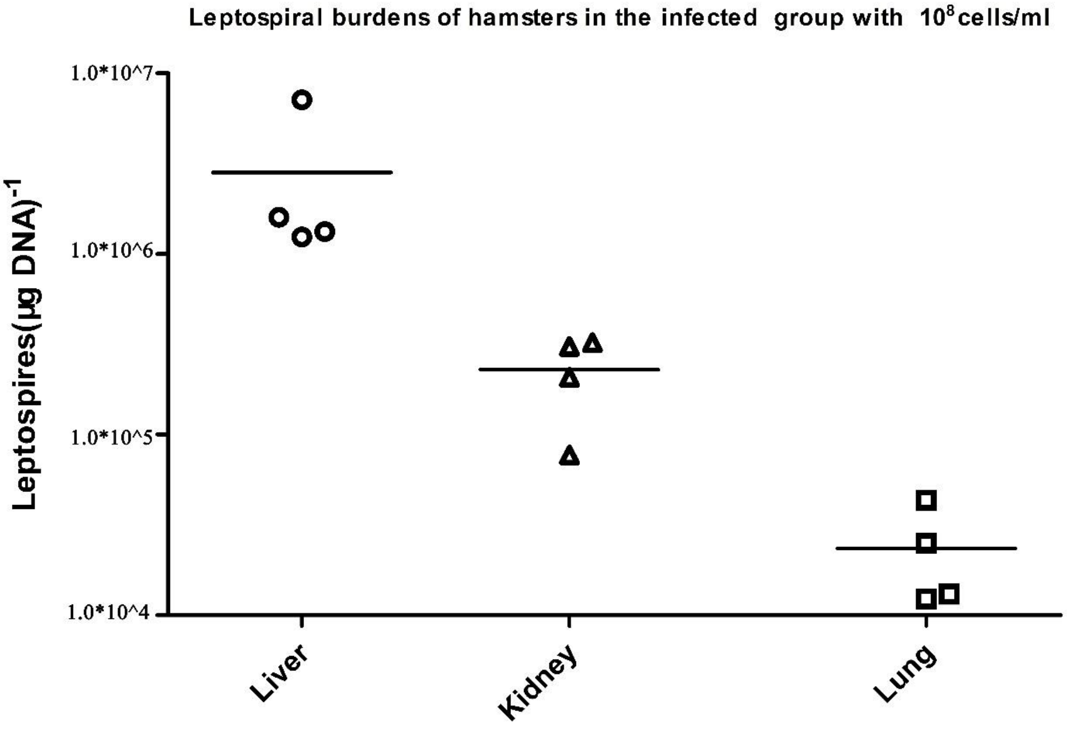

Fig5 Leptospiral burdens in the kidneys, livers, and lungs of hamsters in the infected group with 10^{8} cells/ml
